# *VdSOX1* Negatively Regulates *Verticillium dahliae* Virulence via Enhancing Effector Expression and Suppressing Host Immune Responses

**DOI:** 10.3390/jof11080576

**Published:** 2025-08-01

**Authors:** Di Xu, Xiaoqiang Zhao, Can Xu, Chongbo Zhang, Jiafeng Huang

**Affiliations:** 1 Key Laboratory of Oasis Agricultural Pest Management and Plant Protection Resources Utilization, College of Agriculture, Shihezi University, Shihezi 832000, China; xudi@stu.shzu.edu.cn (D.X.); 16699046676@163.com (C.Z.); 2 Xinjiang Academy of Agricultural and Reclamation Sciences, Shihezi University, Shihezi 832000, China; m18299076107@163.com

**Keywords:** *Verticillium dahliae*, sarcosine oxidase, fungal pathogenicity, effectors expression, suppressing immunity

## Abstract

The soil-borne fungal pathogen *Verticillium dahliae* causes devastating vascular wilt disease in numerous crops, including cotton. In this study, we reveal that *VdSOX1*, a highly conserved sarcosine oxidase gene, is significantly upregulated during host infection and plays a multifaceted role in fungal physiology and pathogenicity. Functional deletion of *VdSOX1* leads to increased fungal virulence, accompanied by enhanced microsclerotia formation, elevated carbon source utilization, and pronounced upregulation of effector genes, including over 50 predicted secreted proteins genes. Moreover, the *VdSOX1* knockout strains suppress the expression of key defense-related transcription factors in cotton, such as WRKY, MYB, AP2/ERF, and GRAS families, thereby impairing host immune responses. Transcriptomic analyses confirm that *VdSOX1* orchestrates a broad metabolic reprogramming that links nutrient acquisition to immune evasion. Our findings identify *VdSOX1* as a central regulator that promotes *V. dahliae* virulence by upregulating effector gene expression and suppressing host immune responses, offering novel insights into the molecular basis of host–pathogen interactions and highlighting potential targets for disease management.

## 1. Introduction

*V. dahliae* is a soil-borne fungal pathogen that causes Verticillium wilt, a destructive vascular disease affecting over 200 plant species, including cotton, tomato, and olive [1,2]. Its pathogenicity is driven by a sophisticated arsenal of virulence factors, enabling it to breach plant defenses, colonize vascular tissues, and survive for years in soil through melanized microsclerotia [3,4]. The molecular mechanisms underpinning its pathogenicity involve a complex interplay of genes regulating microsclerotia formation, host penetration, effector secretion, and immune suppression [5]. Recent studies have identified numerous pathogenicity-related genes [5], yet their functional diversity and synergistic roles remain incompletely understood.

Microsclerotia development and melanization are critical for long-term survival and infection initiation. The high-osmolarity glycerol (HOG) pathway components VdHog1 and VdPbs2 serve as key regulators that coordinately control melanin biosynthesis and microsclerotia development [6,7,8]. Similarly, the C_2_H_2_ transcription factor VdMsn2 and the patatin-like phospholipase VdPLP negatively regulate microsclerotia production but promote virulence [9,10]. Melanin biosynthesis depends on the polyketide synthase gene *VdPKS1* and the laccase gene *VdLac1*; however, disrupting these genes impairs melanization without affecting microsclerotia formation [11,12]. Conversely, the cAMP-dependent kinase VdPKAC1 and the G protein β subunit VGB suppress microsclerotia formation while remaining essential for pathogenicity [13,14].

Pathogens establish infection through specialized adhesion structures and cell wall-degrading enzymes, which facilitate host penetration and subsequent invasive growth. The tetraspan transmembrane protein VdSho1, a homolog of Saccharomyces cerevisiae Sho1, acts as an osmosensor and mediates hyphopodium formation and root penetration [15], while the cellophane surface-induced gene *VdCSIN1* regulates infection via the cAMP pathway [16]. Septin ring organization, which is critical for invasive hyphal growth, requires *VdSep5* and the NADPH oxidase components VdNoxB and VdPls1 for *V. dahliae* colonization [17,18]. Cell wall-degrading enzymes (CWDEs) such as the pectate lyase VdPEL1 and the cellulase VdEg-1 facilitate tissue maceration [19,20]. The sucrose non-fermenting kinase VdSNF1 modulates pectinase activity, linking carbon metabolism to virulence [21].

Effector-mediated immune suppression is central to *V. dahliae* success. Small cysteine-rich proteins (SCPs), including VdSCP27, VdSCP113, and VdSCP126, trigger plant cell death via ROS burst while evading immune recognition [22]. The necrosis-inducing protein PevD1 disrupts cotton defense by interacting with host GhPR5 [23], whereas the glycoside hydrolases VdEG1 and VdEG3 act as pathogen-associated molecular patterns (PAMPs) yet subvert immunity through carbohydrate-binding modules [24]. Additionally, the necrosis and ethylene-inducing protein VdNLP and the isochorismatase VdICSH1 disrupt salicylic acid signaling, thereby suppressing plant defense responses to promote infection [25,26].

Transcription factors (TFs) orchestrate virulence programs. The Zn_2_-Cys_6_ binuclear cluster TF Vdpf regulates melanin biosynthesis and hyphal growth [27], while VdFTF1 (fungal-specific TF) is indispensable for cotton infection [28]. The bZIP TF VdAtf1 mediates nitrogen metabolism and nitrosative stress resistance [29], and VdHapX controls iron homeostasis under nutrient-limited xylem conditions [30]. The MADS-box TF *VdMcm1* maintains cell wall integrity, critical for vascular colonization [31].

Metabolic adaptation ensures survival in hostile host environments. Thiamine biosynthesis genes (*VdThi4*, *VdTHI20*) and transporters (*VdThit*) are vital for conidiation and systemic infection [32,33]. Iron acquisition via VdFreB (ferric reductase) and VdHapX enables growth under iron scarcity [30,34]. The α-oxoglutarate dehydrogenase VdOGDH links the tricarboxylic acid cycle to virulence, highlighting metabolic flexibility as a virulence strategy [35].

Horizontal gene transfer (HGT) and trans-kingdom RNA silencing further exemplify evolutionary innovation. The glucosyltransferase VdGT2 and the LysM effector *VDAG_05180*, acquired via HGT, enhance host-specific virulence [36,37]. Conversely, host-induced gene silencing (HIGS) targeting VdAK (adenylate kinase) or VdILV2 (acetolactate synthase) disrupts fungal metabolism, demonstrating bidirectional RNAi warfare [38,39].

Sarcosine oxidase (EC 1.5.3.1, SOX) is a flavin-containing oxidase capable of oxidizing both secondary and tertiary amines [40]. It serves as the sole key enzyme in the sarcosine metabolic pathway and can be classified into monomeric and polymeric forms [41]. Monomeric sarcosine oxidases (MSOX) are commonly found in fungi, actinomycetes, and bacteria. The term “SOX” originates from the extensively studied MSOX of the genus Bacillus, the first characterized member of the sarcosine oxidase family [42]. Sarcosine oxidase is widely distributed across various organisms, including animals, plants, and microorganisms. It has been detected in animal and plant tissues, such as kidney tissue and *Arabidopsis thaliana*, as well as in bacterial genera like *Bacillus*, *Corynebacterium*, *Streptomyces*, *Arthrobacter*, and *Pseudomonas* [43,44,45,46,47,48,49]. Beyond its metabolic role, SOX is widely applied as a diagnostic enzyme reagent [48]. It is employed in detecting creatinine levels in human serum or urine, serving as a crucial indicator of renal excretory function and playing a significant role in medical diagnostics [50]. Additionally, research has demonstrated that certain microorganisms harboring sarcosine oxidase, such as *Bacillus sphaericus*, can degrade glyphosate via the carbon-phosphorus (C-P) cleavage pathway [51], highlighting its potential applications in bioremediation.

Recent RNA-seq analyses have provided deeper insights into the transcriptional dynamics during microsclerotia biogenesis. Comparative transcriptomic profiling of *V. dahliae* cultures producing melanized microsclerotia (MS+) versus non-producing cultures (NoMS) revealed over 200 differentially expressed genes (DEGs) [52]. Notably, melanogenesis-associated genes, including tetrahydroxynaphthalene reductase (344-fold upregulation) and scytalone dehydratase (231-fold upregulation), were significantly induced under MS+ conditions, underscoring their critical roles in melanin deposition and survival structure maturation [52]. Additionally, a conserved 48.8 kb melanin biosynthetic gene cluster was identified in *V. dahliae*, housing polyketide synthases (PKSs), laccases, and transcription factors such as Pig1 and CMR1, which coordinate pathway regulation and melanin polymerization. These findings highlight the transcriptional complexity underlying microsclerotial development, with nearly 50% of DEGs encoding hypothetical proteins, suggesting unexplored molecular mechanisms in survival structure formation [52].

Despite significant advances, the molecular mechanisms underlying the pathogenicity of *V. dahliae* remain incompletely understood. In this study, we identified a sarcosine oxidase gene (*VdSOX1*) in *V. dahliae*, which was significantly upregulated during host infection (unpublished data). Here, we characterize *VdSOX1*, a SOX homolog in *V. dahliae*, and investigate its role in microsclerotia formation, effector expression, and host immune evasion. Through a combination of genetic knockout, transcriptomic analysis, and plant infection assays, we demonstrate that *VdSOX1* functions as a key regulator of fungal virulence. Our findings provide novel insights into *V. dahliae* pathogenesis and highlight potential targets for disease management strategies.

## 2. Results

### 2.1. VdSOX1 Encodes a Sarcosine Oxidase and Is Highly Conserved Within the Genus Verticillium

**Figure 1:**
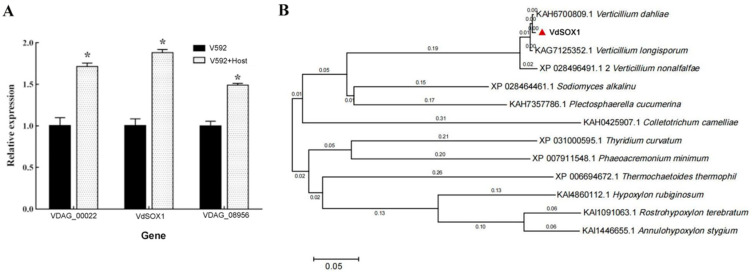
Identification of *VdSOX1*. (**A**) Histogram of the relative expression of *VDAG_00022*, *VdSOX1*, and *VDAG_08956* genes after host induction. Asterisks indicate statistically significant differences (* *p* < 0.05), and error bars represent the standard error of three replicates. (**B**) Phylogenetic analysis of *VdSOX1* and its homologous proteins in *V. dahliae*. The homologous sequences of *VdSOX1* were downloaded from NCBI (https://www.ncbi.nlm.nih.gov/) accessed on 16 September 2023). The full-length protein sequences of *VdSOX1* and its homologous genes were aligned using the neighbor-joining method to construct the phylogenetic tree. The bootstrap replication number was set to 1000. The numbers on the branches indicate genetic divergence, and the scale bar represents 0.05 distance units.

To identify pathogenicity-related genes in *V. dahliae*, RNA-seq was performed on strain V592 treated with or without cotton root. Among the differentially expressed genes, *VDAG_00022*, *VDAG_08956*, and *VdSOX1* showed elevated expression, with RT-qPCR analysis further confirming *VdSOX1* as the most highly upregulated (Figure 1A). To characterize the sequence features of *VdSOX1*, we amplified and sequenced the gene from the V592 strains. Bioinformatic analysis revealed that *VdSOX1* encodes a sarcosine oxidase containing a DAO domain, which belongs to the FAD-dependent oxidoreductase family (Figure S1). To assess the evolutionary conservation of *VdSOX1* among fungi, we retrieved homologous amino acid sequences from the NCBI database and constructed a phylogenetic tree (Figure 1B). The tree demonstrated *VdSOX1* clusters within a clade comprising KAH6700809.1 (*V. dahliae*), KAG7125352.1 (*V. longisporum*), and XP_028496491.1 (*V. nonalfalfae*), with sequence similarity exceeding 90%. Notably, *VdSOX1* exhibited >50% similarity with the remaining fungal homologs (Figure 1B). These findings indicated that *VdSOX1* is highly conserved, particularly within the genus *Verticillium*.

### 2.2. VdSOX1 Exhibits Tissue-Specific Expression and Is Induced by Infection and Sarcosine/Glyphosate

**Figure 2:**
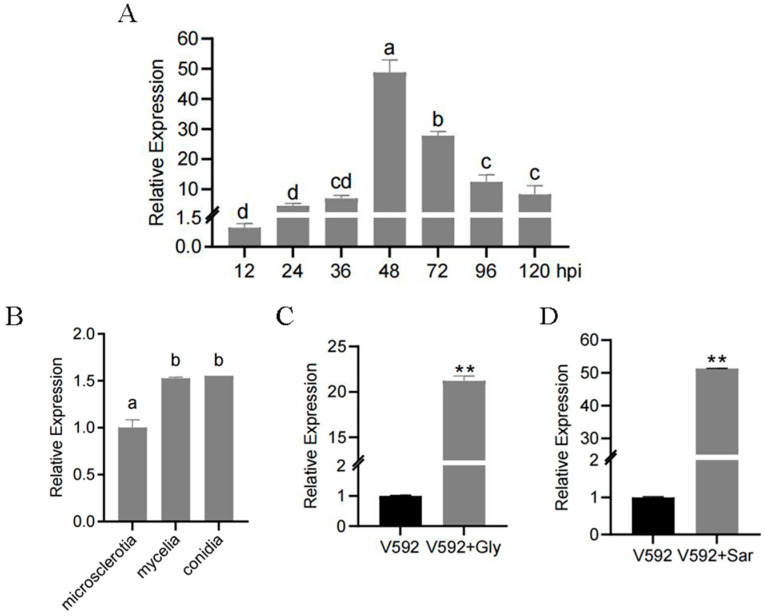
Transcriptional expression of *VdSOX1* gene in *V. dahliae*. (**A**) RT-qPCR analysis of the expression of the *VdSOX1* gene in *V. dahliae* at 12, 24, 36, 48, 72, 96, and 120 h post-infection of cotton. β–tubulin was used as the internal reference gene, with the expression level of *VdSOX1* at 12 h post-infection serving as the control group. Different letters indicate significant differences between samples (*p* < 0.05), and error bars represent the standard error of three replicates. (**B**) RT-qPCR was used to detect the expression of the *VdSOX1* gene in microsclerotia, mycelia, and conidia of *V. dahliae*, with β–tubulin as the internal reference gene. Different letters indicate significant differences among samples (*p* < 0.05), and error bars represent the standard error of three replicates. (**C**) RT-qPCR was used to detect the expression of the *VdSOX1* gene after induction by glyphosate (Gly, 2 g/L) for 48 h. The asterisks indicate statistically significant differences (**, *p* < 0.01), and the error bars represent the standard error of three replicates. (**D**) RT-qPCR was used to detect the expression of the *VdSOX1* gene after induction by sarcosine (Sar, 15 g/L) for 48 h. Asterisks indicate statistically significant differences (**, *p* < 0.01), and error bars represent the standard error of three replicates.

To assess *VdSOX1* expression during *V. dahliae* infection of cotton, we collected infected cotton roots at 12, 24, 36, 48, 72, 96, and 120 h post-inoculation (hpi) and performed RT-qPCR analysis (Figure 2A). The data demonstrated that *VdSOX1* was strongly induced during infection, implying a potential functional importance in the *V. dahliae*–cotton interaction. Furthermore, to investigate the tissue-specific expression pattern of *VdSOX1* in *V. dahliae*, we analyzed its transcript levels in conidia, microsclerotia, and hyphae using RT-qPCR. The results revealed that *VdSOX1* expression was significantly higher in hyphae and conidia than in microsclerotia (Figure 2B), suggesting a tissue-specific role.

*VdSOX1*, which carries a DAO domain, encodes a sarcosine oxidase (Figure S1). Sarcosine oxidase, a key enzyme in the sarcosine degradation pathway, also plays a role in glyphosate metabolism. To investigate whether *VdSOX1* responds to these substrates, we treated *V. dahliae* conidia with glyphosate or sarcosine and analyzed gene expression by RT-qPCR. The results revealed significant upregulation of *VdSOX1* following both glyphosate (Figure 2C) and sarcosine treatments (Figure 2D). These data supported our hypothesis that *VdSOX1* may participate in the degradation pathways of sarcosine and glyphosate.

### 2.3. VdSOX1 Modulates Growth, Sporulation, Microsclerotia Formation, and Virulence in V. dahliae

To generate *VdSOX1* knockout strains, we constructed a gene knockout vector containing the upstream/downstream flanking sequences and a hygromycin phosphotransferase (HPT) selection cassette. Using homologous recombination and *Agrobacterium*-mediated transformation (AMT), we obtained ten transformants. Two stable knockout mutants (*Vdsox1-1* and *Vdsox1-2*) were confirmed through PCR-based genomic validation and RT-qPCR analysis at both DNA and RNA levels (Figure S2, primers are listed in Table S1). For functional complementation, we introduced a *VdSOX1* expression cassette into the *VdSOX1-1* background via AMT, yielding two complemented strains (*EC-sox-1* and *EC-sox-2*) (Figure S2B).

**Figure 3:**
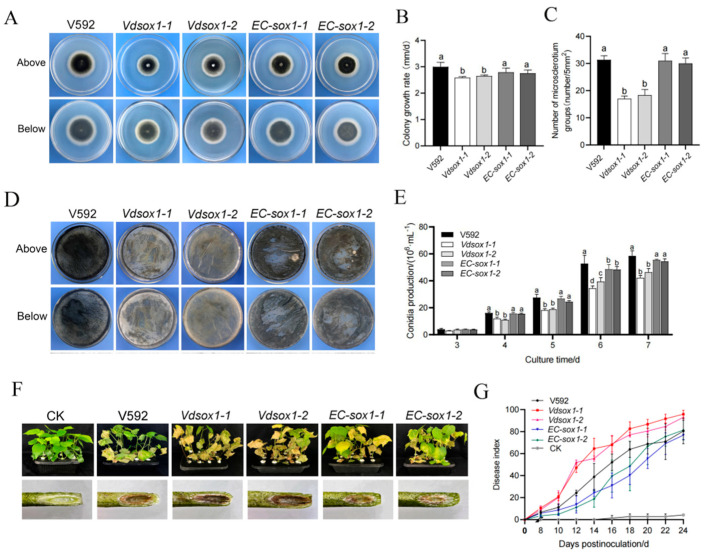
Biological characteristics and pathogenicity of *VdSOX1* mutant strains. (**A**) Phenotypic characteristics of the front and back of colonies of V592, knockout strains (*Vdsox1-1*, *Vdsox1-2*), and complemented strains (*EC-sox1-1*, *EC-sox1-2*) after 15 days of culture on PDA medium. (**B**) Determination of colony growth rates of each strain on PDA mediums, calculated by the formula: Colony growth rate = (average colony diameter on day 9 − Average colony diameter on day 5)/4. Different letters represent significant differences between samples (*p* < 0.05), and error bars indicate the standard error of three replicates. (**C**) Statistics of the number of microsclerotia in every 5 mm field of vision. Different letters represent significant differences between samples (*p* < 0.05), and error bars indicate the standard error of three replicates. (**D**) Microsclerotia production of each strain after 14 days of culture on BMM-deficient medium, with front and back observation images. (**E**) Quantification of sporulation of each strain in Czapek’s liquid medium after shaking culture for 3 d, 4 d, 5 d, 6 d, and 7 d using a hemocytometer under an optical microscope. Different letters indicate significant differences among samples (*p* < 0.05), and error bars represent the standard error of three replicates. (**F**) Symptoms of cotton plants and the degree of vascular browning observed 30 days after inoculation with each strain. (**G**) Disease index of cotton from 8 to 24 dpi with each strain, with error bars representing the standard error of three replicates.

To investigate the functional role of *VdSOX1* in *V. dahliae*, we compared colony morphology, growth rate, sporulation, and microsclerotia production among the wild-type strain V592, *VdSOX1* knockout strains (*Vdsox1-1* and *Vdsox1-2*), and complemented strains (*EC-sox-1* and *EC-sox-2*) on PDA medium and BMM medium. While no apparent differences in colony morphology were observed, the knockout strains displayed significantly reduced growth rates, sporulation capacity, and microsclerotia production compared to both V592 and the complemented strains (Figure 3A–E). Microscopic analysis further confirmed that the conidiophore structure of the *VdSOX1* knockout strains remained intact (Figure S3), indicating that the observed sporulation defect was not attributable to abnormal conidiophore development. Taken together, these findings established *VdSOX1* as a key regulator governing hyphae growth, sporulation, and microsclerotia formation in *V. dahliae*, while being dispensable for conidiophore morphogenesis.

To further investigate the role of *VdSOX1* in *V. dahliae* pathogenicity, we conducted infection assays using the susceptible cotton cultivar Junmian No. 1. Cotton plants were inoculated with *VdSOX1* knockout strains, complemented strains, and wild-type V592. Disease assessment at 24 days post-inoculation (dpi) revealed that the *VdSOX1* knockout strains exhibited significantly enhanced virulence compared to both complemented strains and V592 controls, as evidenced by higher disease indices (Figure 3F,G). Consistent with the disease phenotype, quantitative analysis of fungal biomass demonstrated a substantial increase in *VdSOX1* knockout strain colonization within cotton stem tissues relative to wild-type strains (Figure S4). By contrast, the complemented strains displayed disease symptoms and colonization levels comparable to V592. These findings collectively demonstrated that *VdSOX1* functions as a negative regulator of virulence in *V. dahliae*.

### 2.4. VdSOX1 Negatively Regulates Carbon Metabolism in V. dahliae

**Figure 4:**
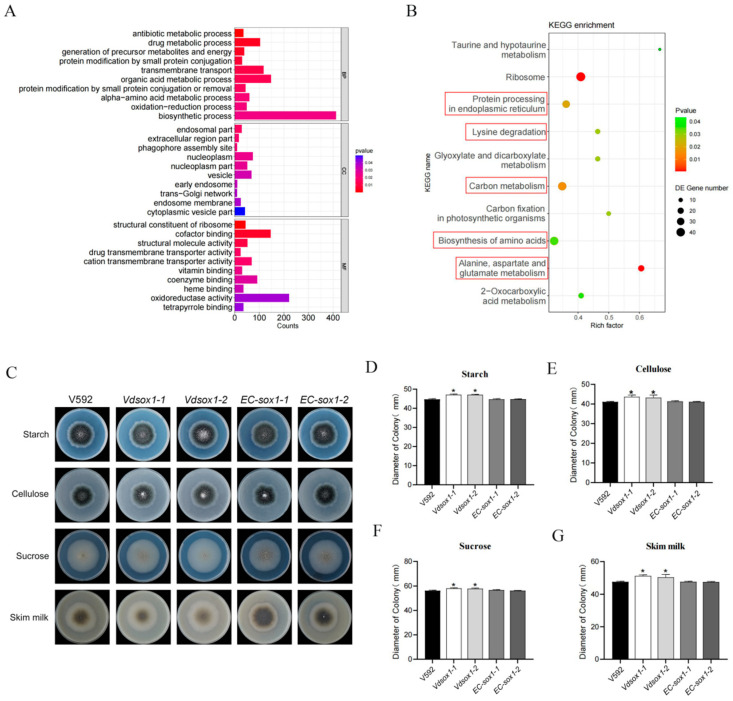
*VdSOX1* gene regulates carbon metabolism in *V. dahliae*. (**A**) Gene Ontology (GO) classification of differentially expressed genes (DEGs) in V592 and *VdSOX1* knockout strains. BP, CC, and MF represent biological process, cellular component, and molecular function, respectively. The *p*-value indicates the statistical significance between variables, and the *x*-axis represents the number of enriched genes. (**B**) Kyoto Encyclopedia of Genes and Genomes (KEGG) classification of DEGs in V592 and *VdSOX1* knockout strains. The *p*-value indicates the statistical significance between variables, and the number represents the count of enriched genes. (**C**) Growth status of each strain cultured on different carbon source media for 15 days. (**D**–**G**) Colony diameters of each strain after being cultured for 15 days on media with starch, cellulose, sucrose, and skim milk as the sole carbon sources, respectively. The asterisk indicates a statistically significant difference (*, *p* < 0.05), and the error bars represent the standard error of three replicates.

To investigate the regulatory role of the *VdSOX1* gene in *V. dahliae*, we performed comparative transcriptomic analysis by RNA sequencing of conidia from both the *VdSOX1* knockout strains and the wild-type V592 strains. *VdSOX1* induced 2484 differentially expressed genes (DEGs), with 1216 upregulated genes and 1268 downregulated genes (Figure S5). It was verified by RT-qPCR that the change trend was consistent with the results obtained by transcriptomic analysis (Figure S6). GO enrichment analysis of the upregulated differentially expressed genes (DEGs) demonstrated significant enrichment in multiple functional categories. In biological processes (BP), DEGs were predominantly associated with biosynthetic processes, organic acid metabolic processes, transmembrane transport, and drug metabolism. For cellular components (CC), DEGs were mainly localized to the nucleoplasm, vesicular structures, and related compartments. Regarding molecular functions (MF), DEGs exhibited strong enrichment in oxidoreductase activity, cofactor binding, coenzyme binding, and cation transmembrane transporter activity (Figure 4A). Furthermore, KEGG pathway enrichment analysis revealed that DEGs were primarily involved in ribosome biogenesis, protein processing in the endoplasmic reticulum, carbon metabolism, and amino acid biosynthesis, among other key metabolic pathways. (Figure 4B) Collectively, these results indicated that *VdSOX1* serves as a critical regulator in *V. dahliae*, orchestrating diverse physiological processes including cellular biosynthesis, metabolic flux, transmembrane transport, and redox balance.

Given that the invasion and colonization of *V. dahliae* in host plants are closely associated with carbon source utilization, and considering that our transcriptome data revealed significant enrichment of differentially expressed genes (DEGs) in carbon metabolic processes (with 3 genes downregulated and 35 genes upregulated; Figure S7), we sought to determine how *VdSOX1* deletion affects carbon metabolism. To assess this, we cultured *VdSOX1* knockout strains, complementary strains, and V592 on Czapek solid media containing starch, cellulose, sucrose, or skim milk powder as sole carbon sources. After 14 days of growth, colony diameters were measured. Notably, the *VdSOX1* knockout strains exhibited significantly larger colonies compared to V592 across all carbon sources (Figure 4C–G). These findings demonstrated that *VdSOX1* acts as a negative regulator of *V. dahliae*’s ability to decompose and utilize starch, cellulose, sucrose, and skim milk powder.

### 2.5. VdSOX1 Gene Negatively Promotes Effector Expression to Enhance Pathogenicity

**Figure 5:**
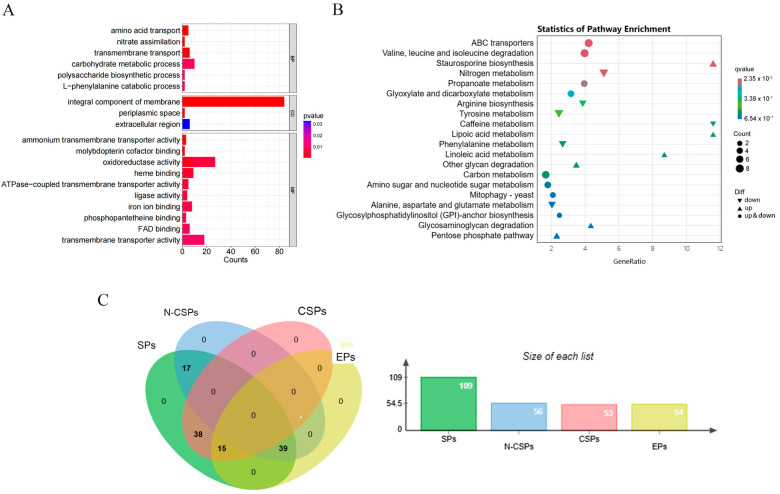
*VdSOX1* gene regulates the expression of pathogenicity-related genes in *V. dahliae*. (**A**) Gene Ontology (GO) classification of DEGs after cotton induction in V592 and *VdSOX1* knockout strains. BP, CC, and MF represent biological process, cellular component, and molecular function, respectively. The *p*-value indicates the statistical significance between variables, and the horizontal axis represents the number of enriched genes. (**B**) Kyoto Encyclopedia of Genes and Genomes (KEGG) classification of DEGs after cotton induction in V592 and *VdSOX1* knockout strains. The *p*-value indicates the statistical significance between variables, and the numbers represent the number of enriched genes. (**C**) Venn diagram of the number of secreted proteins encoded by upregulated DEGs. SPs, Secreted proteins; CSPs, Classical secreted proteins; N-CSPs, Non-classical secreted proteins; EPs, Effector proteins. The data are shown in Table S3.

To investigate the regulatory mechanism by which *VdSOX1* knockout enhances pathogenicity in *V. dahliae*, we simulated host–pathogen interactions by putting sterile cotton roots into the spore suspension of *VdSOX1* knockout strains and V592 strains for 48 h. Subsequently, we collected conidia from both strains following this root treatment for comparative transcriptomic analysis. A total of 380 genes were altered between the *VdSOX1* knockout strains and V592 after induction, with 190 upregulated and 190 downregulated (Figure S8). RT-qPCR validation confirmed that the expression trends were consistent with the transcriptome sequencing data (Figure S9). Gene Ontology (GO) enrichment analysis revealed distinct functional patterns among the differentially expressed genes (DEGs). In biological processes (BP), DEGs showed significant enrichment in amino acid transport, nitrate assimilation, transmembrane transport, and carbohydrate metabolic processes. For cellular components (CC), DEGs were predominantly associated with integral membrane components and periplasmic space. Molecular function (MF) analysis demonstrated enrichment in ammonium transmembrane transporter activity, pterin-type molybdenum cofactor binding, oxidoreductase activity (particularly involving heme and iron ion binding), and ATPase-coupled transmembrane transporter activity (Figure 5A). KEGG pathway analysis further revealed significant enrichment in branched-chain amino acid degradation (valine, leucine, and isoleucine), ABC transporter systems, staurosporine biosynthesis, nitrogen metabolism, and propanoate metabolism (Figure 5B). Our findings revealed that *VdSOX1* knockout modulates *V. dahliae*’s growth, development, and virulence by reprogramming the expression of key genes involved in metabolic and biosynthetic processes.

Notably, among the 308 upregulated DEGs identified in cotton root-treated samples, 109 genes (35.4%) encoded predicted effector proteins. These included 53 classical secreted proteins (CSPs) and 56 non-classical secreted proteins (N-CSPs) (Figure 5C). These findings strongly suggested that *VdSOX1* knockout enhances *V. dahliae* pathogenicity, at least in part, through the significant upregulation of effector genes, particularly those encoding both classical and non-classical secreted effector proteins.

### 2.6. VdSOX1 Knockout Enhances Host Susceptibility by Downregulating Cotton Resistance-Related Genes

**Figure 6:**
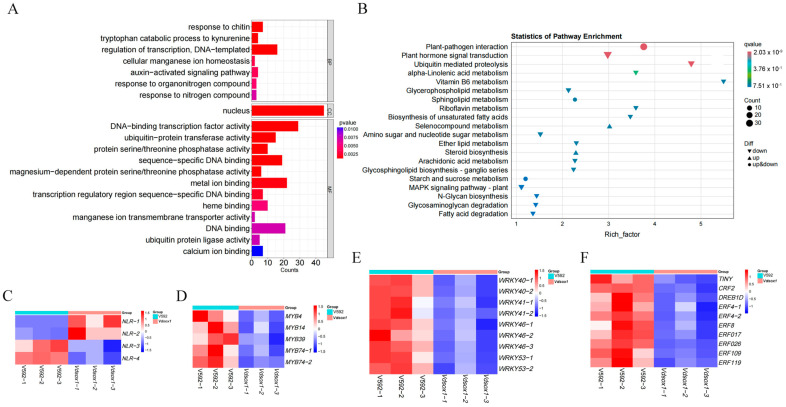
*VdSOX1* gene regulates the expression of disease resistance-related genes in host plants. (**A**) Gene Ontology (GO) classification of differentially expressed genes (DEGs) in cotton after inoculation with V592 and *VdSOX1* knockout strains. BP, CC, and MF represent biological process, cellular component, and molecular function, respectively. The *p*-value indicates the statistical significance between variables, and the *x*-axis represents the number of enriched genes. (**B**) Kyoto Encyclopedia of Genes and Genomes (KEGG) classification of DEGs in cotton after inoculation with V592 and *VdSOX1* knockout strains. The *p*-value indicates the statistical significance between variables, and the numbers represent the number of enriched genes. (**C**–**F**) Expression of plant resistance-related genes among the DEGs. The heatmaps respectively represent the expression patterns of disease resistance protein-related genes, MYB transcription factors, WRKY transcription factors, and AP2/ERF transcription factors. Red indicates upregulation, while blue indicates downregulation. The data are shown in Table S4.

To further elucidate the mechanism by which *VdSOX1* knockout enhances host susceptibility, we inoculated cotton roots with spore suspensions of the *VdSOX1* knockout strains and the wild-type V592 strain for 96 h, followed by transcriptome sequencing analysis of the harvested root tissues. RNA-seq data revealed that *VdSOX1* knockout induced significant alterations in the expression of 245 cotton genes, comprising 22 upregulated and 223 downregulated genes (Figure S10). RT-qPCR analysis corroborated the transcriptome data, showing matching expression dynamics (Figure S11). Gene Ontology enrichment analysis demonstrated significant enrichment in several biological processes, including response to chitin, tryptophan catabolism to kynurenine, and regulation of transcription. Molecular function categories were notably enriched for DNA-binding transcription factor activity and ubiquitin-protein transferase activity (Figure 6A). Kyoto Encyclopedia of Genes and Genomes (KEGG) pathway analysis of differentially expressed genes (DEGs) revealed significant enrichment in plant-pathogen interaction pathways, hormone signal transduction, and ubiquitin-mediated proteolysis (Figure 6B).

Further functional characterization of genes within these pathways showed marked downregulation of key transcription factor genes associated with plant disease resistance, including WRKY, MYB, and AP2/ERF families (Figure 6D–F). Additionally, most genes encoding plant disease resistance proteins exhibited predominant downregulation (Figure 6C). These findings collectively suggested that *VdSOX1* knockout compromises cotton’s defense response by suppressing critical immune-related transcriptional networks and pathogen recognition systems.

## 3. Discussion

In recent years, an increasing number of studies have focused on the microsclerotia, hyphae, conidia, and pathogenicity of *V. dahliae*. Current studies have shown that the deletion of most genes affects the growth, development, and pathogenicity of *V. dahliae*. For example, the transcription factor *VdSge1* [53] and the gene *VdMsb* [54], which encodes transmembrane mucin, regulate the production of conidia and microsclerotia. The knockout mutants of *VdNLP1* and *VdNLP2* can promote vigorous growth of the mycelium [55]. The deletion of the mitogen-activated protein kinase *VdPbs2* [6], the two-component stress response regulator gene *VdSkn7* [56], the α-1,6-mannosyltransferase gene *VdOCH1* [57], and the Ada1 subunit gene *VdAda1* [58] all reduce the pathogenicity of *V. dahliae*. Here, we analyzed the role of *VdSOX1*, a sarcosine oxidase gene, which modulates growth, sporulation, microsclerotia formation and virulence in *V. dahliae* (Figure 3).

Transcriptomics is an important research approach that systematically analyzes gene expression profiles and their regulatory networks at the RNA level, thereby revealing organisms’ physiological states and molecular mechanisms. In our study, *VdSOX1* was initially identified through comprehensive RNA-seq analysis, with its expression subsequently validated by reverse transcription quantitative PCR (RT-qPCR) (Figure 1A). Comparative transcriptomic profiling was conducted on strain V592 under two conditions: exposure to cotton root versus untreated control. Among the differentially expressed genes identified, three candidates (*VDAG_00022*, *VDAG_08956*, and *VdSOX1*) demonstrated significant upregulation. Subsequent RT-qPCR analysis confirmed *VdSOX1* as the most dramatically upregulated gene in response to cotton root treatment (Figure 1A). Genetic knockout and pathogenicity assays revealed that *VdSOX1* is an important virulence-related gene in *V. dahliae.* By performing comparative transcriptomic analysis between wild-type and knockout mutant strains, it was possible to identify genes involved in metabolism and pathogenicity that were regulated by the target gene. Transcriptomic comparison between wild-type (WT) and *ΔVdPT1* strains revealed that downregulation of genes associated with carbon metabolism, DNA replication, and amino acid biosynthesis critically contributes to the observed growth impairment in *ΔVdPT1* mutants [59]. In this study, transcriptomic analysis of the *VdSOX1* knockout strains demonstrated that deletion of this gene leads to significant upregulation of key genes in the amino acid biosynthesis pathway of *V. dahliae*. As *VdSOX1* encodes a glycine/D-amino acid oxidase domain known to participate in deamination reactions and regulate amino acid transport/metabolism, this transcriptional reprogramming likely represents a compensatory response to impaired amino acid metabolism (Figure 4B). Concurrently, we observed marked downregulation of genes involved in endoplasmic reticulum (ER)-associated protein processing (Figure 5). This suppression of ER protein homeostasis machinery may critically underlie the attenuated growth and developmental rates observed in the *VdSOX1* knockout strains (Figure 4B).

The homeostatic regulation of carbon metabolism represents a fundamental molecular mechanism underlying the pathogenicity of filamentous fungi. These organisms secrete an array of extracellular hydrolases, including cellulases, hemicellulases, and pectinases, to break down complex polysaccharides into assimilable carbon sources. This catabolic process serves dual critical functions: (1) providing essential energy for fungal growth and reproduction, and (2) supplying precursor metabolites for biosynthetic pathways that support pathogenicity-related processes. Such processes encompass infection structure formation, virulence factor synthesis, and host tissue colonization [60,61]. Notably, genetic studies have revealed important regulators of this metabolic–pathogenic interface. The *ΔVdHP1* knockout mutant demonstrated enhanced radial growth on Czapek–Dox medium containing diverse carbon sources (sucrose, cellulose, and skim milk), concomitant with increased virulence [62]. Similarly, our investigation of *VdSOX1* revealed striking phenotypic consequences of its deletion. The *VdSOX1* knockout strains exhibited markedly improved utilization efficiency for various carbon substrates, including starch, cellulose, sucrose, and skim milk powder (Figure 4C–G). Transcriptomic profiling further demonstrated significant upregulation of carbon metabolic pathway genes in the *VdSOX1* knockout strains (Figure S6). Importantly, pathogenicity assays confirmed substantially enhanced virulence in the knockout strain. These collective findings support a model wherein *VdSOX1* deletion potentiates fungal pathogenicity through metabolic reprogramming (Figure 3E,F). The observed upregulation of carbon metabolic pathways likely enhances nutrient acquisition from host tissues, thereby fueling pathogenic development. This study provides novel insights into the molecular connections between carbon metabolism regulation and virulence in *V. dahliae*.

Pathogens deliver effectors into plant cell to attack the plant immune system and promote infection. Accumulating evidence demonstrates that effectors secreted by *V. dahliae* manipulate plant immune responses to enhance virulence. *Vd6317* directly inhibits the activity of the plant transcription factor *AtNAC53*, thereby suppressing the expression of *AtUGT74E2* and plant defense [63]. The effector protein VdPHB1 promotes *V. dahliae* infection in cotton by inhibiting the activity of cysteine proteases [64]. This finding highlights the pivotal role of *VdSOX1* in orchestrating metabolic reprogramming in the pathogen, significantly enhancing its adaptability to the host environment and consequently improving infection efficiency through coordinated regulation of key biological processes, including secondary metabolite biosynthesis, protein processing, and nutrient metabolism (Figure 5B). Further analysis revealed that the *VdSOX1* knockout strains displayed altered expression of 112 proteins, including 54 effector proteins, all of which were upregulated (Figure 5C). Therefore, we hypothesize that the *VdSOX1* knockouts may enhance pathogenicity toward cotton by upregulating the expression of effectors. In the pathogen–host interaction system, effectors secreted by the pathogen into the host cell inhibit the expression of host defense genes by interfering with host transcriptional regulation, hijacking signaling pathways, and inducing protein degradation and epigenetic modification. For example, VdCE51 interferes with the defense response mediated by the SA signaling pathway by inhibiting the accumulation of GhTRXH2 in the nucleus and reducing the transcriptional expression level of the PR gene [65]. The synthesis of effector proteins requires a large amount of energy and amino acids, and fungi may regulate metabolic allocation through certain genes (such as prioritizing growth rather than effector protein synthesis). After knocking out metabolic allocation regulatory genes, fungi will allocate more resources to the synthesis of effector proteins, especially under infection pressure, the expression of effector proteins will increase significantly to enhance virulence. This regulatory relationship plays a role in T cell-mediated immunity [66].

Transcription factors are key regulators of plant immunity. During the infection process, *V. dahliae* secretes a large number of effectors to regulate plant immunity. For example, VdSCP41 is secreted from fungi and transported into the nucleus of plants, directly targeting the important transcription factors CBP60g and SARD1 of plants, interfering with the activity of transcription factors to inhibit the induction of plant immune-related genes, and helping *V. dahliae* colonization [67]. Our findings demonstrate *VdSOX1* as a negative regulator of *V. dahliae* virulence, mediated through its suppression of critical transcription factor families (WRKY, MYB, and AP2/ERF) associated with pathogenicity (Figure 6D–F).

Our findings regarding carbon metabolism utilization demonstrated that the *VdSOX1* knockout strains exhibited faster growth than wild-type strain V592 on polysaccharide-rich media (e.g., starch, cellulose, sucrose, and skim milk) but showed reduced growth on glucose-based medium (Figure 4C). The observed growth inhibition on PDA may be attributed to its high glucose content. As noted in reference [68], glucose exerts a ‘double-edged sword’ effect: while low to moderate concentrations generally promote growth, high concentrations can inhibit growth due to metabolic by-product accumulation or osmotic pressure changes. Notably, although elevated glucose concentrations enhanced polysaccharide production, they concurrently reduced cell density (OD value), suggesting a shift in carbon flux toward secondary metabolism rather than biomass accumulation. By contrast, the polysaccharides supplemented in our media—composed of diverse monosaccharides rather than pure glucose—appeared to stimulate colony growth more effectively.

Collectively, our findings demonstrate that *VdSOX1* plays a multifunctional role in *V. dahliae* pathogenesis by modulating fungal growth and development through the regulation of microsclerotia formation, conidiation, and carbon source utilization and coordinating virulence during host infection by promoting the expression of fungal effector proteins while simultaneously suppressing plant defense-related genes. These results provide critical insights into the molecular mechanisms governing *V. dahliae*–cotton interactions and highlight *VdSOX1* as a central regulator of both pathogenic development and host-pathogen interplay.

## 4. Materials and Methods

### 4.1. V. dahliae Isolates and Growth Conditions

*V. dahliae* isolate V592 isolated from infected cotton plants in Xinjiang, China [14], was used as the wild-type strain and the knockout mutants were generated from V592 in this study. For the phenotypic characteristics or collecting mycelia, V592 and its derived mutants were grown on potato dextrose agar (PDA) plates supplemented with or without different carbon sources for 15 d at 25 °C, as described elsewhere [69]. To obtain fungal cultures for RNA isolation or to harvest conidia for infection assays, strains of V592 and its derived mutants were grown in liquid Czapek–Dox medium under constant agitation (200 rpm) at 25 °C in complete darkness.

### 4.2. Cloning and Sequence Analysis of VdSOX1 Gene

Fungal DNA was extracted using the Biospin Fungus Genomic DNA Extraction Kit (BioFlux, Hangzhou, China) from fresh fungal mycelia. The conidia were then collected, and fungal RNA was extracted using Trizol reagent (Invitrogen, Carlsbad, CA, USA). cDNA was synthesized using the PrimeScript^®^ RT reagent kit with gDNA eraser (TaKaRa, Osaka, Japan). To determine the *VdSOX1* sequence, PCR fragments were amplified using both genomic DNA and cDNA as templates (Table S1). The resulting PCR products were then purified, cloned, and sequenced. To identify and retrieve full-length homologs of *VdSOX1*, we performed BLASTP searches against the NCBI protein database (http://www.ncbi.nlm.nih.gov/) accessed on 16 September 2023) using the *VdSOX1* protein sequence as a query. The resulting homologous sequences from other fungal species were then collected for further analysis. Sequence alignment and structural comparison of *VdSOX1* with its homologs were performed using both ESPript 3.0 (https://espript.ibcp.fr/ESPript/cgi-bin/ESPript.cgi, accessed on 20 September 2023) for enhanced visualization of conserved residues and Geneious Prime software (version 2023.0.4) for comprehensive sequence analysis. The phylogenetic analysis was performed using MEGA6.06 software, with trees constructed using the neighbor-joining method and branch support assessed through 1000 bootstrap replicates.

### 4.3. Analysis of Gene Expression Patterns

For sample preparation, the V592 strain was sequentially cultured under three conditions: (1) mycelia were obtained from 5-day PDA plate cultures, (2) conidia were collected from 3-day liquid Czapek–Dox cultures with shaking, and (3) microsclerotia were harvested from 20-day BMM medium cultures (yeast extract 10 g/L, peptone 20 g/L, 1 M buffer of potassium phosphate pH 6.0, 1.34% amino acid-free yeast basic nitrogen source, 0.00004% Biotin, 0.5% methanol).

Total RNA was isolated from mycelia, conidia with or without glyphosate treatment, microsclerotia, or cotton roots harvested at different time points post-inoculation with V592 as well as uninfected roots that served as a negative control. Real-time qPCR was performed using the HiScript II One Step qRT-PCR SYBR Green Kit (Vazyme, Nanjing, China) with the primers listed in Table S1 and the 7500 Fast Real-Time PCR System (Applied Biosystems, Foster City, CA, USA). The expression of *VdSOX1* genes was normalized to that of internal control gene *β-tubulin Gh18S*.

### 4.4. Plasmid Construction and Transformation

To generate the knockout plasmid pGKO-*VdSOX1*, upstream and downstream genomic sequences were amplified with the specific primer pairs listed in Table S1. Both genomic sequences were ligated into a position flanking of the hygromycin resistance cassette of the vector pGKO with Infusion cloning (ClonExpress MultiS One Step Cloning Kit-C113, Nanjing, China) to generate knockout plasmids. The agrobacterium-mediated transformation was conducted as described previously [70]. The knockout mutants were confirmed using the PCR method with the primers *VdSOX1*-full-F/R (Table S1). The complementation plasmid pNEO-*VdSOX1* was generated by inserting the *VdSOX1* genomic sequence (primers listed in Table S1) into an EcoRI/BamHI-digested pNEO vector under the control of a constitutive promoter, using Infusion cloning (ClonExpress II One Step Cloning Kit C112, Nanjing, China). The pNEO-*VdSOX1* plasmid was then transformed into a deletion mutant to produce the complemented mutants. The complemented strains were screened using the antibiotic *G418* and PCR method with the primers *VdSOX1*-full-F/R (Table S1). Finally, all mutants were verified by qRT-PCR analysis, with expression levels normalized to that of the internal control gene *β-tubulin* (GenBank accession: DQ266153).

### 4.5. Phenotype Assays

The colony diameter and conidial production of fungal strains were measured as previously described [11]. For mycelial morphological analysis, knockout mutants and the V592 control strain were point-inoculated at the center of PDA plates (*n* = 5 replicates per strain) and dark-incubated at 25 °C for 3 days. Microscopic examination of hyphal development was subsequently performed as previously described [11]. The microsclerotia formation assay was performed as previously described [69]. The cellophane penetration assay was conducted as previously described [69] with minor modifications. Fungal mycelia were inoculated onto minimal medium (MM) plates overlaid with sterile cellophane membranes using sterile toothpicks, followed by incubation in complete darkness at 22 °C for 3 days. After this initial growth period, plates were maintained under the same conditions for an additional 5 days to allow full hyphal penetration, after which penetration efficiency was assessed through microscopic examination and photographic documentation.

### 4.6. The Cotton Infection Assays

Cotton plants (cv. Junmian No. 1, Bayingolhan Mongolian Autonomous Prefecture Agricultural Research Institute, Korla, China) were employed in pathogenicity assays to assess the comparative virulence of wild-type strain V592 and transformants. The evaluation was conducted using the root-dip inoculation method, which was performed as previously described [14] with minor modifications. Fungal inoculation was performed on fifth true-leaf stage cotton seedlings with 36 cotton plants by complete root immersion in an aqueous conidial suspension (1.0 × 10^7^ conidia/mL in sterile distilled water) for 1 h, with 200 mL applied per hydroponic chamber (three chambers per treatment as biological replicates). After 1 h inoculation, the conidial suspension was replaced with fresh hydroponic solution. Beginning on day 8 dpi until 28 dpi, disease severity was assessed using the standardized cotton Verticillium wilt rating scale, with disease index calculated as previously described [69]. At 30 dpi, cotton stems were longitudinally dissected to examine vascular browning, with representative samples photographed for documentation.

### 4.7. RNA-Sequencing

The conidia of *VdSOX1* knockout strains and V592 strain cultured in liquid medium were collected. The conidia of *VdSOX1* knockout strains and V592 strain were collected after 48 h of cotton root induction. Cotton was inoculated with conidial suspension of *VdSOX1* knockout strains and V592 strain, and cotton roots were collected after 96 h of culture. Samples were sent to Biomarker Technologies Co., Ltd. (Beijing, China). Each strain was tested for 3 replicates.

### 4.8. Read Mapping and Differential Expression Analysis

The sequencing data were assessed for quality using FastQC (v0.11.9), followed by trimming with Trimmomatic (v0.32). The processed reads were then mapped to the *V. dahliae* reference genome (ASM975710v1) using HISAT2 (v2.2.1) and further processed with SAMtools (v1.13). Gene-level read counts were obtained using HTSeq (v0.11.3) with the following parameters: -f bam -r name -s no -t exon -i transcript_id -m intersection-nonempty. All statistical analyses were performed using R (version 4.3.2; R Foundation for Statistical Computing) within the RStudio (R version 4.3.2) Server environment. For differential expression analysis, we utilized the DESeq2 package (v1.40.2). Data visualization was implemented using ggplot2 (v3.4.4), pheatmap (v1.0.12), RColorBrewer (v1.1.3), and gplots (v3.1.3). Additional analyses were conducted using amap (v0.8-19) for distance calculations, ggrepel (v0.9.4) for improved label placement, and dplyr (v1.1.3) for data manipulation. Parallel processing was enabled through BiocParallel (v1.34.0). For data exploration, including principal component analysis, correlation analysis, and GO term enrichment, we identified differentially expressed genes (DEGs) using relaxed thresholds (|log_2_ (fold change)| ≥ 1 and adjusted *p* < 0.01) to capture a broader landscape of transcriptional changes.

### 4.9. Exploratory Data Analysis

Gene expression patterns induced by the *VdSOX1* gene or cotton were visualized using customized implementations of the ggplot2 (v3.4.4) and pheatmap (v1.0.12) packages in R. We identified and analyzed DEG genes through clustering analysis performed using Venny 2.1 (https://bioinfogp.cnb.csic.es/tools/venny/, accessed on 15 January 2024). Gene Ontology (GO) enrichment analysis was subsequently conducted using a web-based tool (http://www.bioinformatics.com.cn/?p=1, accessed on 20 January 2024). The data were analyzed using IBM SPSS Statistics for Windows (Version 20.0), and one-way analysis of variance (ANOVA) was employed for data analysis (* *p* < 0.05, ** *p* < 0.01).

## Data Availability

The original contributions presented in this study are included in the article/Supplementary Materials. Further inquiries can be directed to the corresponding authors.

## Supplementary Materials

The following supporting information can be downloaded at: https://www.mdpi.com/article/10.3390/jof11080576/s1, Figure S1. The structure diagram of the *VdSOX1* amino acid sequence; Figure S2. Construction and screening of the *VdSOX1* knockout vector; Figure S3. Observation of conidiophore morphology of various strains under an optical microscope; Figure S4. Measurement of fungal biomass in cotton plants after inoculation with various strains; Figure S5. The number of differential genes in V592 strain and *VdSOX1* knockout strains; Figure S6. RT-qPCR detection of differential genes in V592 strains and *VdSOX1* knockout strains. Figure S7. The expression of genes related to carbon metabolism in DEGs; Figure S8. The number of differential genes between V592 and *VdSOX1* knockout strains after cotton induction; Figure S9. RT-qPCR detection of differential genes between V592 and *VdSOX1* knockout strains after cotton induction. Figure S10. The number of differential genes in cotton after inoculation with V592 and *VdSOX1* knockout strains; Figure S11. RT-qPCR detection of differential genes in cotton after inoculation with V592 and *VdSOX1* knockout strains. Table S1. Primers used in this study; Table S2. Differential genes related to carbon metabolism; Table S3. Secreted protein-related differential genes; Table S4. Disease resistance related differential genes.
