# Supplementary material for: VdSOX1 Negatively Regulates Verticillium dahliae Virulence via Enhancing Effector Expression and Suppressing Host Immune Responses
Source: J Fungi (Basel). 2025 Aug 1;11(8):576. doi: 10.3390/jof11080576 (PMC12387551; doi:10.3390/jof11080576)
Supplement: Supplementary file 1 [file jof-11-00576-s001.zip › Supplementary Figure.pdf]

Supplementary Materials

VdSOX1 Negatively Regulates *Verticillium dahliae* Virulence via Enhancing Effectors Expression and Suppressing Host Immune Responses

Di Xu[1,†] Xiaoqiang Zhao[1,†,*], Can Xu[2], Chongbo Zhang[1], Jiafeng Huang[1,*]

1 Key Laboratory of Oasis Agricultural Pest Management and Plant Protection Resources Utilization, College of Agriculture, Shihezi University, Shihezi 832000, China; xudi@stu.shzu.edu.cn (D. X.); m18299076107@163.com (C. X.); 16699046676@163.com (C. Z)

2 Xinjiang Academy of Agricultural and Reclamation Sciences, Shihezi University, Shihezi 832000, China;

[†] These authors contributed equally to this work

* Correspondence: zhaoxq2025@163.com (X. Z.); hjf@shzu.edu.cn (J. H.)

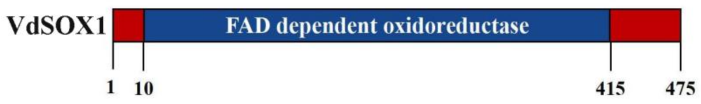

Figure S1. The structure diagram of *VdSOX1* amino acid sequence. The numbers represent amino acid lengths

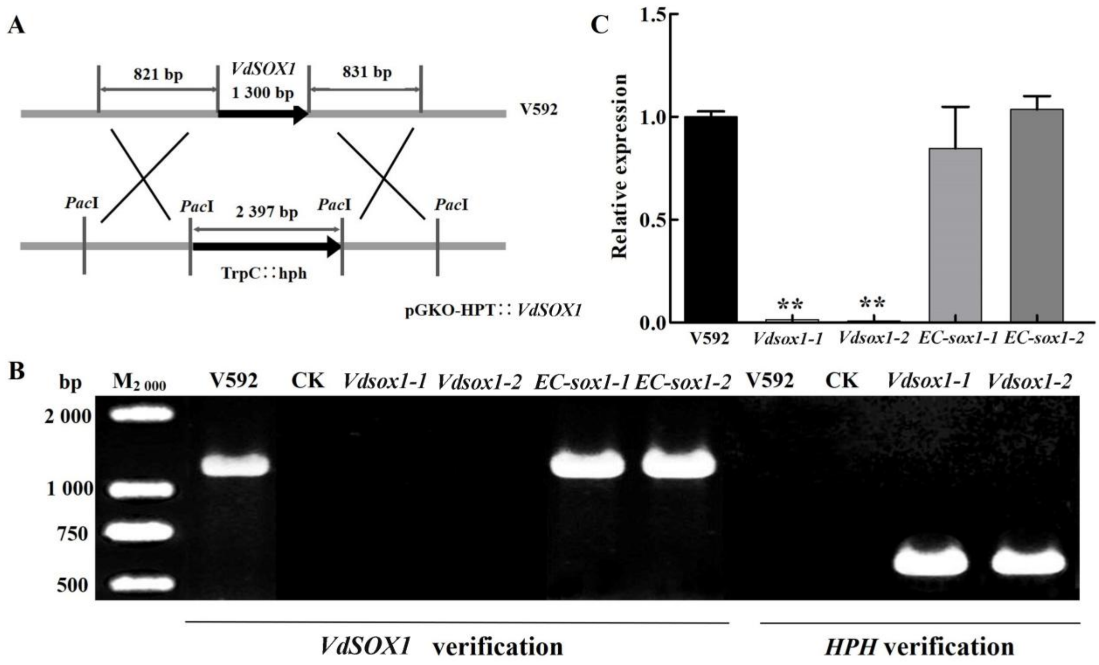

Figure S2. Construction and screening of the *VdSOX1* knockout vector. (A) Schematic diagram of the construction strategy for the *VdSOX1* knockout vector. (B) PCR verification of the *VdSOX1* knockout and complemented strains. M2 000 is the marker, V592 is the wild-type strain, and CK is the negative control. (C) Transcriptional level verification of the *VdSOX1* knockout and complemented strains. Data were analyzed for significant differences using Dunnett's test, ** indicates extremely significant differences compared to V592 ($P < 0.01$), and error bars represent the standard error of three replicates.

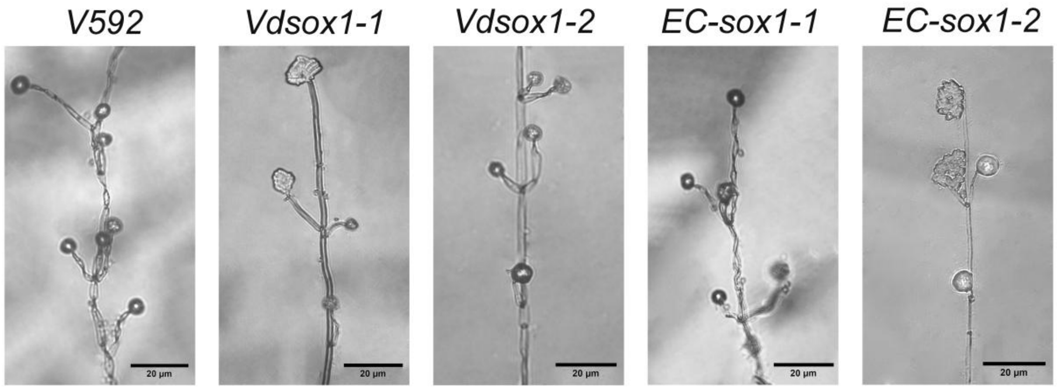

Figure S3. Observation of conidiophore morphology of various strains under an optical microscope, Bar = 20 μm.

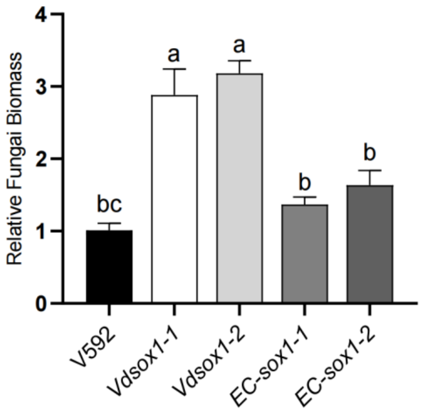

Figure S4. Measurement of fungal biomass in cotton plants after inoculation with various strains. Different letters indicate significant differences among samples (*P < 0.05*), and error bars represent the standard error of three replicates.

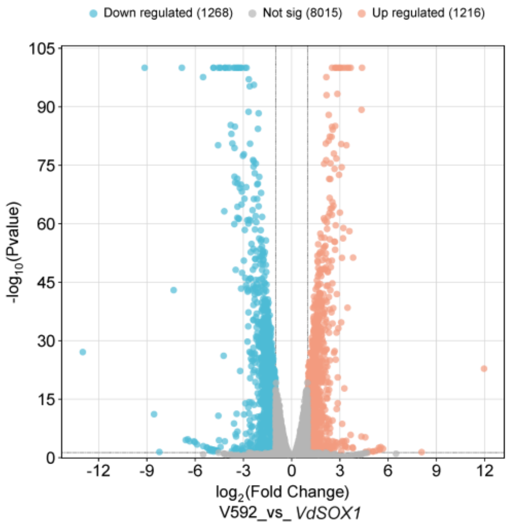

Figure S5. The number of differential genes in V592 strains and *VdSOX1* knockout strains.

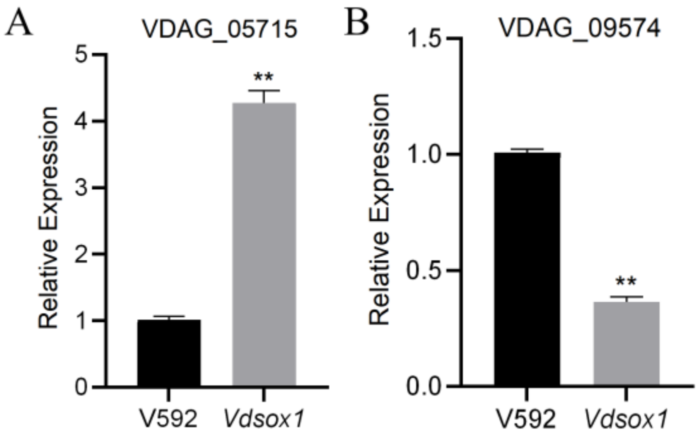

Figure S6. RT-qPCR detection of differential genes in V592 strains and *VdSOX1* knockout strains. (A) Hypothetical protein gene VDAG_05715. (B) FAD-dependent oxidoreductase gene VDAG_09574. Data were analyzed for significant differences using Dunnett's test, ** indicates extremely significant differences compared to V592 ($P < 0.01$), and error bars represent the standard error of three replicates.

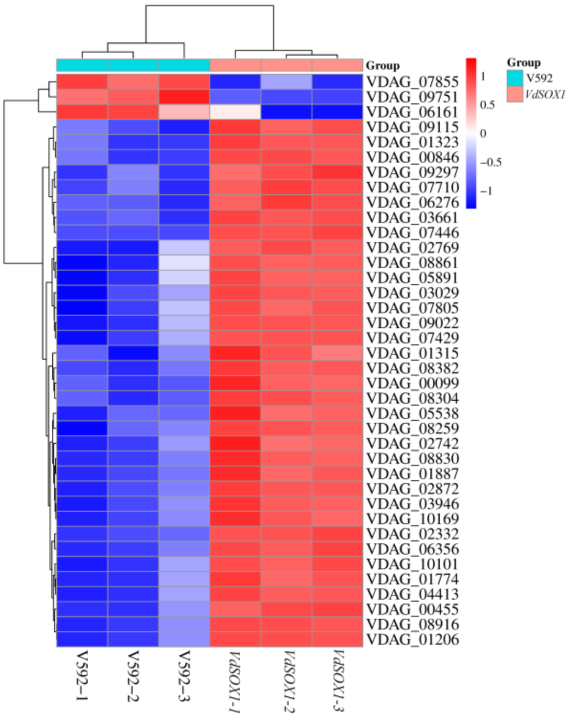

Figure S7. The expression of genes related to carbon metabolism in DEGs. Red represents up-regulation and blue represents down-regulation. The data are shown in Table S2.

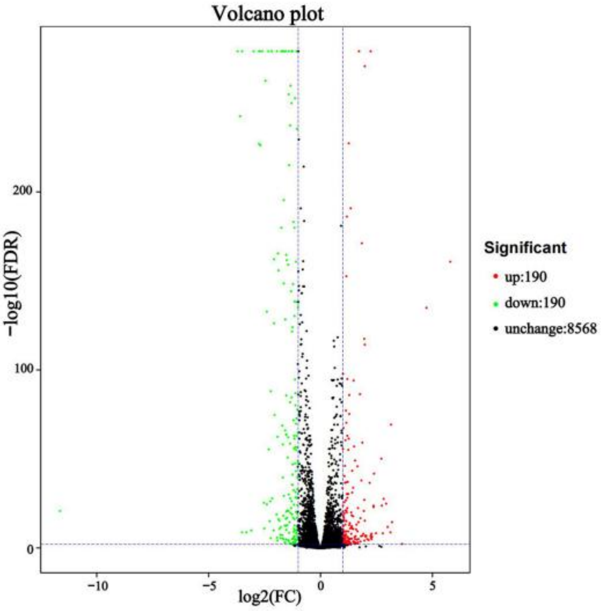

Figure S8. The number of differential genes between V592 and *VdSOX1* knockout strains after cotton induction.

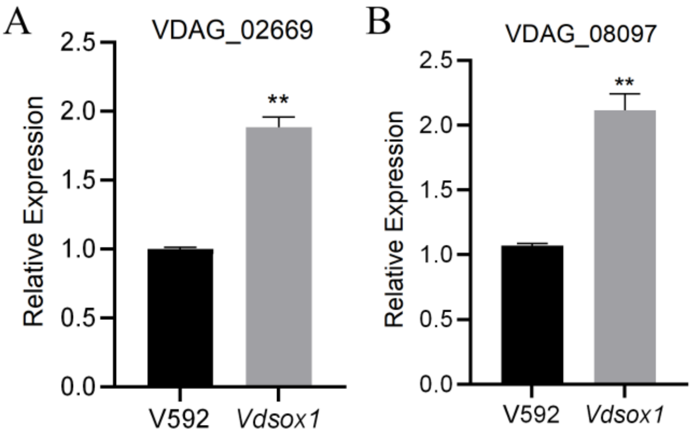

Figure S9. RT-qPCR detection of differential genes between V592 and *VdSOX1* knockout strains after cotton induction. (A) hypothetical effector protein gene VDAG_02669. (B) Glycosyl hydrolases family 28 gene VDAG_08097. Data were analyzed for significant differences using Dunnett's test, ** indicates extremely significant differences compared to V592 (*P < 0.01*), and error bars represent the standard error of three replicates.

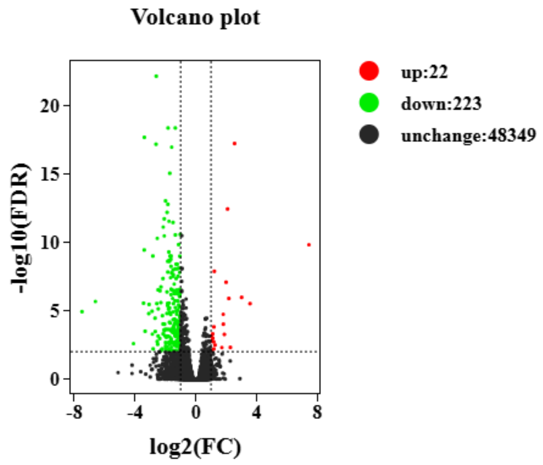

Figure S10. The number of differential genes in cotton after inoculation with V592 and *VdSOX1* knockout strains.

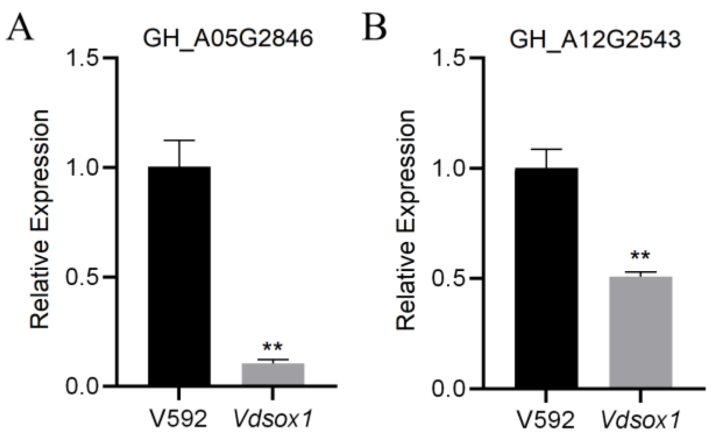

Figure S11. RT-qPCR detection of differential genes in cotton after inoculation with V592 and *VdSOX1* knockout strains. (A) Phloem protein gene GH_A05G2846. (B) WRKY transcription factor gene GH_A12G2543. Data were analyzed for significant differences using Dunnett's test, ** indicates extremely significant differences compared to V592 ($P < 0.01$), and error bars represent the standard error of three replicates.
